# Ruxolitinib shows activity against Hodgkin lymphoma but not primary mediastinal large B-cell lymphoma

**DOI:** 10.1186/s12885-019-6303-z

**Published:** 2019-11-10

**Authors:** Seok Jin Kim, Dok Hyun Yoon, Hye Jin Kang, Jung Yong Hong, Ho Sup Lee, Sung Yong Oh, Ho-Jin Shin, Jee Hyun Kong, Jun Ho Yi, Kana Sakamoto, Young Hyeh Ko, Jooryung Huh, Seung-Sook Lee, Kengo Takeuchi, Dong-Yeop Shin, Cheolwon Suh, Won Seog Kim

**Affiliations:** 1 Division of Hematology-Oncology, Department of Medicine, Samsung Medical Center, Sungkyunkwan University School of Medicine, 81 Irwon-ro, Gangnam-gu, Seoul, 06351 South Korea; 2 Department of Oncology, Asan Medical Center, University of Ulsan College of Medicine, Seoul, South Korea; 3 Department of Internal Medicine, Korea Cancer Center Hospital, Korea Institute of Radiological and Medical Sciences, Seoul, South Korea; 4 Department of Internal Medicine, Kosin University Gospel Hospital, Busan, South Korea; 5 Department of Internal Medicine, Dong-A University College of Medicine, Busan, South Korea; 6 Department of Internal Medicine, Pusan National University Hospital, Busan, South Korea; 7 Department of Internal Medicine, Wonju Severance Christian Hospital, Wonju, South Korea; 8 Department of Internal Medicine, Chung-Ang University Hospital, Seoul, South Korea; 9 Division of Pathology, The Cancer Institute, Japanese Foundation for Cancer Research, Tokyo, Japan; 10 Pathology Project for Molecular Targets, The Cancer Institute, Japanese Foundation for Cancer Research, Tokyo, Japan; 11 Department of Pathology, Samsung Medical Center, Sungkyunkwan University School of Medicine, Seoul, South Korea; 12 Department of Pathology, Asan Medical Center, University of Ulsan College of Medicine, Seoul, South Korea; 13 Department of Pathology, Korea Cancer Center Hospital, Korea Institute of Radiological and Medical Sciences, Seoul, South Korea; 14 Department of Internal Medicine, Seoul National University Hospital, Seoul, South Korea

**Keywords:** Hodgkin lymphoma, Mediastinal large B-cell lymphoma, JAK2, Ruxolitinib

## Abstract

**Background:**

The upregulated expression of the JAK/STAT pathway promotes tumor growth in Hodgkin lymphoma (HL) and primary mediastinal large B-cell lymphoma (PMBCL). Based on the hypothesis that JAK2 is a therapeutic target, we performed a prospective pilot study using ruxolitinib.

**Methods:**

Relapsed or refractory patients with HL or PMBCL were eligible for this study, and *JAK2* amplification was assessed by fluorescence in situ hybridization. Ruxolitinib was administered orally at a dose of 20 mg twice daily for a 28-day cycle. Treatment was continued for up to 16 cycles or until progressive disease or intolerability. The primary objective was to assess the overall disease control rate comprising complete response (CR), partial response (PR), or stable disease (SD).

**Results:**

We analyzed 13 HL patients and six PMBCL patients. All responders (one CR, five PR, and one SD) had HL whereas all cases of PMBCL progressed after first or second cycle. The disease control rate for HL was 54% (7/13) with median response duration of 5.6 months. *JAK2* amplification was present in six of nine patients tested (four HL, two PMBCL), and three of these HL patients showed PR (*n* = 2) or SD (*n* = 1). None of the three HL patients shown to not have *JAK2* amplification responded to ruxolitinib. Most treatment-related adverse events were grade 1 or 2 and manageable.

**Conclusions:**

Ruxolitinib has single-agent activity against HL but does not act against PMBCL with or without *JAK2* amplification.

**Trial registration:**

The study population was patients who had relapsed or refractory HL or PMBCL, and patients were registered for our pilot study after providing written informed consent between November 2013 and November 2015 (CilinicalTrials.gov: NCT01965119).

## Background

Hodgkin lymphoma (HL) is a chemotherapy-sensitive malignancy, and the majority of cases can be cured by multi-agent combination chemotherapy such as ABVD (doxorubicin, bleomycin, vinblastine and dacarbazine). However, relapse still occurs, especially, in patients with advanced disease even after complete remission. Once patients fail to be cured by first-line chemotherapy, they usually show dismal prognosis [1]. Primary mediastinal large B-cell lymphoma (PMBCL), a subtype of non-Hodgkin lymphoma has similar clinical features to HL in terms of involved sites and clinical features [2]. Like HL, the treatment outcome of PMBCL was improved by the addition of rituximab to CHOP (cyclophosphamide, doxorubicin, vincristine and prednisolone), however, a substantial number of patients failed to be cured after becoming refractory to conventional chemotherapy [3]. Thus, effective treatment strategy for relapsed or refractory disease has been required for these disease entities. The Janus kinase 2 (JAK2) leads to activation of the signal transducer and activator of transcription (STAT) pathway, which can also be activated by cytokines from the tumor microenvironment and by a gain of chromosome 9p [4]. HL is frequently associated with a 9p24.1 genomic amplification that includes the *JAK2* locus and with a cytokine-enriched tumor microenvironment [5, 6]. Thus, activation of the JAK2/STAT signaling pathway could promote tumor growth in HL [7, 8]. JAK2 activation also appears frequently in primary mediastinal large B-cell lymphoma (PMBCL), because PMBCL shares molecular features with HL [9, 10]. Thus, we hypothesized that the inhibition of JAK2 could be an effective treatment strategy for these patients, and performed a prospective pilot study with ruxolitinib, a potent and selective JAK1/JAK2 inhibitor, for treatment of patients with relapsed or refractory HL and PMBCL [11].

## Methods

### Study design

This pilot study (NCT01965119) was designed to evaluate the efficacy and safety of ruxolitinib in patients with relapsed or refractory HL and PMBCL. Patients aged 18 years or older with at least one measurable lesion with a greatest transverse diameter ≥ 1.5 cm were eligible after having received at least two prior therapies including salvage therapies and/or autologous stem cell transplantation. In addition, patients should have adequate organ function as defined by the following criteria: serum aspartate transaminase (AST) and serum alanine transaminase (ALT) ≤ 2.5 × local laboratory upper limit of normal (ULN), or AST and ALT less than or equal to 5 × ULN if liver function abnormalities are due to underlying malignancy; total serum bilirubin ≤1.5 × ULN; absolute neutrophil count (ANC) ≥ 1500/μL; platelets ≥100,000/μL; hemoglobin ≥9.0 g/dL; serum calcium ≤12.0 mg/dL and serum creatinine ≤1.5 × ULN. However, patients who had lymphomatous involvement of the central nervous system were excluded. Patients previously undergoing allogeneic stem cell transplantation and patients with uncontrolled active infection were also excluded. A pathology review was performed by the Korean Lymphoma Pathology Review Board after enrollment was completed. Ruxolitinib was administered orally to participants at a dose of 20 mg twice daily for a 28-day cycle. Treatment was continued for up to 16 cycles or until progressive disease or intolerability. The primary objective was to assess the overall disease control rate, defined as comprising complete response (CR), partial response (PR), or stable disease (SD). Response evaluation was conducted by the investigators according to the 2007 Revised International Working Group Response Criteria for Malignant Lymphoma [12]. The first assessment was performed within a week of the expected start date of the third treatment cycle using computed tomography (CT) and positron emission tomography/computed tomography (PET/CT) of the neck, chest, abdomen, and pelvis. Subsequent response evaluations were performed during the fourth, eighth, twelfth, and sixteenth cycles by CT. For apparently new lesions, PET/CT was performed to confirm disease progression. Toxicity was assessed and adverse events were graded using the National Cancer Institute Common Terminology Criteria for Adverse Events (version 4.0). We received official approval from the Korean Food and Drug Administration, and each study site obtained approval from their local institutional review board. All patients gave written informed consent prior to study participation.

### Assessment of JAK2 amplification

For assessment of *JAK2* amplification by fluorescence in situ hybridization (FISH), unstained slides were subjected to hybridization with bacterial artificial chromosome (BAC) clone-derived DNA probes for *JAK2*. The BAC clones used will be provided upon request. The hybridized slides were then counterstained with 4′,6-diamidino-2-phenylindole and examined with a BX51 fluorescence microscope (Olympus, Tokyo, Japan) to count the number of signals to evaluate *JAK2* amplification in tumor cells.

### Survival analysis

Assessment of disease and survival status took place every 3 months as per institutional standards of care and thereafter until study closure or withdrawal of consent. Overall survival (OS) and progression-free survival (PFS) were calculated from the first date of ruxolitinib administration to final follow-up or death from any cause, or the date of disease progression, respectively. The last survival status update was on July 30, 2018. Survival was estimated based on Kaplan–Meier curves and compared using the log-rank test.

## Results

### Patients

**Fig. 1:**
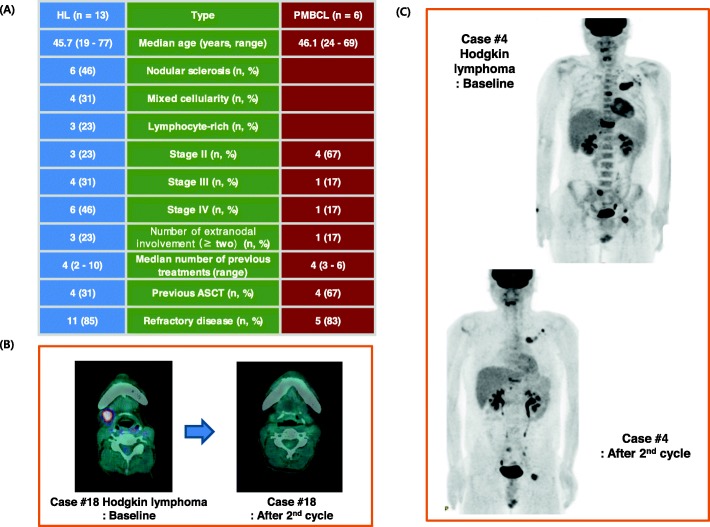
**a** Comparison of clinical characteristics of patients between Hodgkin lymphoma and primary mediastinal large B-cell lymphoma at the time of enrollment; **b** One case of Hodgkin lymphoma achieved complete response; **c** One patient with Hodgkin lymphoma achieving stable disease after the second cycle also showed improved disease status

In total, 20 patients were enrolled (median age: 43 years, range: 19–77 years) between November 2013 and November 2015 from eight hospitals of the Consortium for Improving Survival of Lymphoma (CISL). However, the central pathology review during the final analysis reported that one reported case of HL was actually EBV-associated lymphoproliferative disease. Thus, we analyzed 13 patients with HL and six patients with PMBCL. All patients with HL initially received ABVD chemotherapy whereas all but one of the patients with PMBCL received R-CHOP chemotherapy as first-line treatment. The demographic and clinical characteristics of the patients with HL and PMBCL at the time of enrollment did not differ except for the number of patients with stage IV disease; 84% (16/19) of the patients had refractory disease (Fig. 1a).

### Response to ruxolitinib

**Fig. 2:**
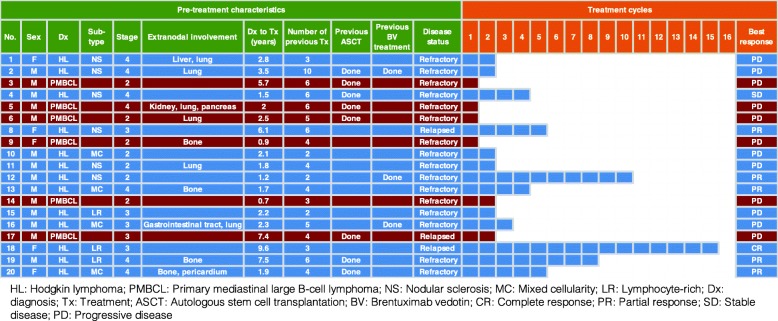
Summary of 19 patients receiving ruxolitinib treatment

Among 13 patients with HL, five patients achieved PR and one patient showed CR (Fig. 1b). The other patient with HL achieved SD after the second cycle of treatment. He showed clinically improvement of symptoms and disappearance of tumor lesions although some lesions were persistent (Fig. 1c). The median duration of response was 5.6 months in six responders including one CR and five cases with PR (95% confidence interval [CI]: 0.01–12.1 months). However, all PMBCL patients rapidly progressed after the first or second cycle of treatment (Fig. 2). The 74-year old female patient achieving CR had stage III, lymphocyte-rich HL at the time of enrollment (Patient number 18, Fig. 2). Although she had experienced two episodes of relapse after she was initially treated with ABVD, she maintained her CR status until the 15th cycle. Of the three patients with HL who had previously received brentuximab vedotin, one patient achieved a PR and maintained this response until the tenth cycle (Fig. 2). As a result, the overall disease control rate for HL was 54% (7/13) although the overall disease control rate for all participants was 36.8% (7/19).

### *JAK2* amplification

**Fig. 3:**
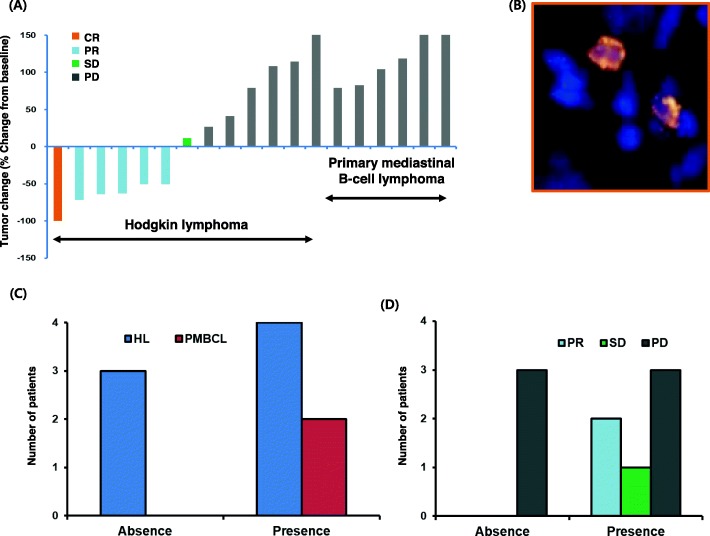
**a** Waterfall plot demonstrating percent change from baseline in target tumor dimensions (best response, *n* = 19); **b** A representative image of *JAK2* amplification; **c** *JAK2* amplification is present in six patients: four Hodgkin lymphoma and two primary mediastinal large B-cell lymphoma; **d** Three of six patients with *JAK2* amplification achieved partial response or stable disease, whereas three Hodgkin lymphoma patients without *JAK2* amplification showed disease progression

The waterfall plot of this study demonstrated all of the responders to ruxolitinib were HL patients (Fig. 3a). FISH analysis of *JAK2* amplification was performed in nine patients for whom tissue samples were available to analyze the association of *JAK2* amplification with response to ruxolitinib. Out of nine patients, *JAK2* amplification was demonstrated in six patients (four HL, two PMBCL, Fig. 3b, c). Three of these six patients, all of whom had HL, showed PR or SD. However, the three HL patients without *JAK2* amplification showed PD (Fig. 3d). Although the number of patients was too small for statistical analysis, HL patients with *JAK2* amplification seemed to have a high probability of responding to ruxolitinib, while the two PMBCL patients with *JAK2* amplification did not respond to ruxolitinib (Fig. 3d).

### Survival outcome and toxicities

**Fig. 4:**
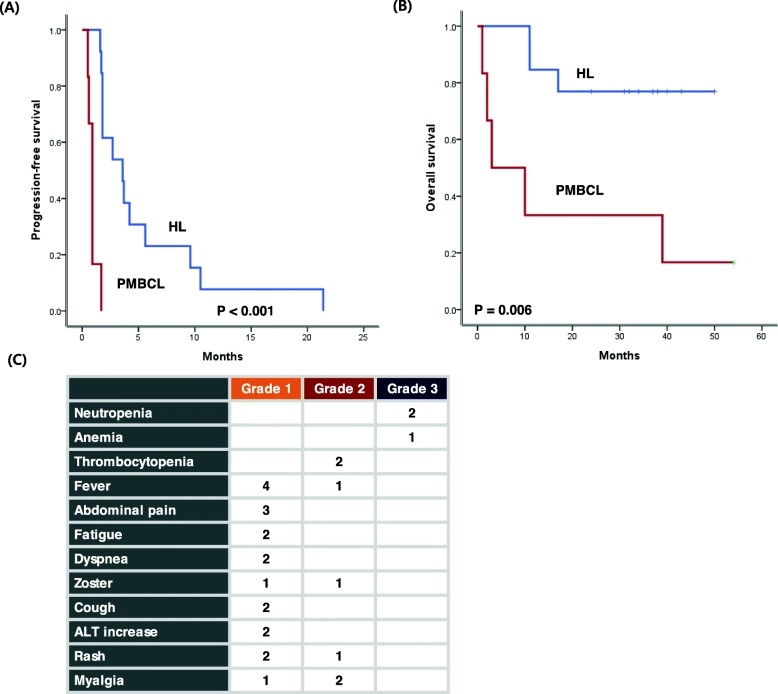
**a**, **b** The progression-free survival and overall survival of Hodgkin lymphoma patients were significantly longer than those of primary mediastinal large B-cell lymphoma patients; **c** Summary of treatment-related adverse effects

The median PFS of HL patients (3.6 months, 95% CI: 1.4–5.8 months) was longer than that of PMBCL patients (0.9 months, 95% CI: 0.72–1.08 months, Fig. 4a). The median OS of HL patients was not reached within the median follow-up of 37.0 months (95% CI: 32.5–41.5 months), and was thus also much better than that of PMBCL (3.0 months, 95% CI: 0.0–12.6, Fig. 4b). Treatment-related adverse events were reported in 14 patients (73.6%), however, most events were grade 1 or 2 (Fig. 4c). Grade 3 neutropenia and anemia were observed in three patients, all of whom recovered. As a result, there was no dose-modification according to the occurrence of hematologic and non-hematologic toxicities.

## Discussion

In our study, ruxolitinib showed acceptable efficacy for relapsed/refractory HL patients. However, only one patient achieved CR, and the duration of response was relatively short. This outcome was similar to that of a phase II study of ruxolitinib treatment for relapsed/refractory HL patients. In the study, best overall response rate was 18.8% (6/32 patients), and the median duration of response was 7.7 months. The survival analysis reported the median PFS of 3.5 months (95% CI: 1.9–4.6), and the median OS of 27.1 months (95% CI: 14.4–27.1) similar to that of our study [13]. Notably, our study showed ruxolitinib was not effective in PMBCL patients although the number was relatively small.

These different outcomes of HL and PMBCL patients were not consistent with previous preclinical studies [14, 15]. Actually, the JAK2 selective inhibitor, fedratinib decreased the cellular proliferation of HL and PMBCL cell lines, and reduced tumor growth in murine xenograft models of cHL and MLBCL with *9p24.1/JAK2* amplification [14]. The anti-tumor effect of ruxolitinib was also demonstrated in both HL and PMBL cells and xenograft models [15]. Thus, ruxolitinib inhibited the growth of both HL and PMBCL cells and increased programmed cell death. In addition, ruxolitinib inhibited tumor progression improving survival of HL as well as PMBCL xenograft mice. These anti-tumor effects of ruxolitinib against PMBCL in pre-clinical studies were opposite to the poor outcome of PMBCL patients in our study. This discrepancy between the results of pre-clinical studies and that of our clinical study could not be clearly explained due to the lack of robust evidence supporting poor outcome of PMBCL patients. However, there might be several possible explanations for that. First, the influence of JAK2 signaling on the growth of tumor cells in human might be different from in vitro and in vivo models, and this might be related with different outcome of ruxolitinib in HL and PMBCL of this pilot study. Second, JAK/STAT signaling is one of various signaling pathways contributing to the survival and aggressiveness of tumor cells such as NF-kB and T-cell exhaustion. Indeed, the gain of mutation in *REL* related with NF-kB pathway was reported up to 75% in PMBCL patients suggesting the important role of NF-kB in PMBCL [16, 17]. Thus, the influence of JAK2 inhibition on the growth of tumor cells in PMBCL might be less than that of HL. Actually, we evaluated the presence of *JAK2* amplification in patients enrolled onto our study to find a biomarker for predicting the response to ruxolitinib. Although the number of patients in our study was small, our FISH analysis showed a possible association of *JAK2* amplification with response to ruxolitinib in HL, because three of four HL patients shown to have a *JAK2* mutation responded to treatment. In a previous phase II study of ruxolitinib analyzing 12 patients for *JAK2* amplification, specific *JAK2* amplification was found in only one patient who achieved PR [13]. However, our PMBCL cases with *JAK2* amplification did not respond, suggesting that PMBCL might be less dependent than HL on JAK2 activation. This result might be consistent with relatively lower activity of PD1 inhibitor in PMBCL than HL where amplification of chromosome 9p.24.1 induced PD1 ligand (PD-L1) expression [18, 19]. Third, the protective effect of cytokines might be related with clinical outcomes different from pre-clinical studies because several cytokines, including interleukin-6 were shown to protect *JAK2V617F* mutant cells from treatment with a JAK2 inhibitor [20]. As JAK2 activation could lead to PD-L1 expression resulting in T-cell anergy and immune escape, ruxolitinib could inhibit this T-cell exhaustion in tumor microenvironment [21]. However, ruxolitinib also could inhibit natural killer (NK) cell activity resulting in decrease of NK-cell mediated immune surveillance against tumor cells [22]. Therefore, this opposite effect of ruxolitinib might influence the outcomes of our study. Furthermore, it is not certain whether our dosage was appropriate for inhibiting JAK2 in HL and PMBCL tumor cells because we used the dosage recommended for myeloproliferative neoplasms. A phase I/II study of ruxolitinib in acute myeloid leukemia reported the tolerability of ruxolitinib at doses up to 200 mg twice daily [23]. Given the tolerable toxicity profiles in our study, dosage escalation might improve the efficacy profile of ruxolitinib in HL and PMBCL patients.

## Conclusions

In conclusion, this pilot study suggested that ruxolitinib might have single-agent activity against HL at the current dosage, especially in case of patients with *JAK2* amplification. However, ruxolitinib might not be effective against PMBCL regardless of *JAK2* amplification. Considering the biological rationale for the use of JAK2 inhibitor as a treatment of HL, further study should be warranted to explore the optimal usage of JAK2 inhibitor such as combined approach of JAK2 inhibitor with brentuximab vedotin, nivolumab or pembrolizumab.

## Data Availability

All data generated or analyzed during this study are available from the corresponding author on reasonable request.

## Abbreviations

ABVD: Doxorubicin, bleomycin, vinblastine and dacarbazine
CR: Complete response
CT: Computed tomography
HL: Hodgkin lymphoma
OS: Overall survival
PD: Progressive disease
PFS: Progression-free survival
PMBCL: Primary mediastinal large B-cell lymphoma
PR: Partial response
R-CHOP: Rituximab, cyclophosphamide, doxorubicin, vincristine and prednisone
SCT: Stem cell transplantation
SD: Stable disease
